# NAD^+^-Dependent Lysine Acetylation Regulates Glucose Uptake and Fatty Acid Oxidation in Cardiomyocytes

**DOI:** 10.3390/metabo15100636

**Published:** 2025-09-23

**Authors:** Ettore Vanni, Christophe Montessuit

**Affiliations:** 1 Pole of Cardiovascular Research, Institute of Clinical and Experimental Research, Catholic University of Louvain, B-1200 Brussels, Belgium; ettore.vanni@uclouvain.be; 2 Department of Pathology and Immunology, Faculty of Medicine, University of Geneva, CH-1205 Geneva, Switzerland

**Keywords:** heart, cardiomyocytes, metabolism, post-translational modification, protein acetylation, NAD metabolites

## Abstract

Background/Objectives: Stimulation of glucose uptake in response to ischemic stress is important for cardiomyocyte post-ischemic function and survival. In the diabetic myocardium chronically exposed to an excess of circulating lipids, this mechanism is impaired, making the myocardium more sensitive to ischemia–reperfusion injury (IRI). In vitro studies have shown that exposure to fatty acids (FAs) reduces basal and stimulated glucose uptake in cardiomyocytes. Preliminary results indicate reduced NAD^+^ levels and increased protein lysine acetylation in FA-exposed cardiomyocytes. This study aims to investigate whether intracellular NAD^+^ reduction is responsible for FA-induced increase in protein acetylation and impaired glucose uptake. Methods: Primary rat cardiomyocytes were chronically treated with the sirtuin deacetylase inhibitor nicotinamide (NAM) in absence of FAs to induce protein acetylation. Conversely, we replenished NAD^+^ concentration using nicotinamide riboside (NR) to induce protein deacetylation in FA-exposed cardiomyocytes. Results: Similar to FA exposure, NAM treatment increased protein acetylation and impaired metabolic-stress-stimulated glucose uptake in cardiomyocytes. In contrast, NR supplementation reduced protein acetylation and improved metabolic-stress-stimulated glucose uptake in FA-exposed cardiomyocytes. Neither NAM nor NR influenced insulin-stimulated glucose uptake. Both NAM and FAs induced hydroxyacyl-CoA dehydrogenase trifunctional enzyme subunit α (HADHA) acetylation on lysine residues K^166^ and K^214^ and enhanced palmitate oxidation. Conversely, NR treatment induced HADHA deacetylation and reduced palmitate uptake and oxidation in FA-exposed cardiomyocytes. Conclusions: In cardiomyocytes, protein hyperacetylation, resulting from either FA exposure or sirtuin inhibition, impairs metabolic-stress-stimulated glucose uptake and is associated with increased FA oxidation.

## 1. Introduction

Type 2 diabetes mellitus (T2DM) is known to increase both the incidence and severity of myocardial infarction. The inability of the diabetic myocardium to adequately stimulate glucose uptake during metabolic stress could be one important reason for the worse outcome of ischemia/reperfusion (I/R) [1,2]. However, the cellular mechanisms responsible for the defective metabolic stress response in diabetic cardiomyocytes remain poorly understood.

T2DM is characterized by hyperglycemia, hyperinsulinemia, and increased circulating concentrations of fatty acids (FAs) and triglycerides. Excessive provision and oxidation of FAs increases the mitochondrial ratios of both acetyl-CoA/CoA and NADH/NAD^+^, favoring protein acetylation over deacetylation [3]. Lysine acetylation is a reversible post-translational modification process where lysine acetyltransferases (KAT) transfer the acetyl moiety from acetyl-CoA to the amino groups of lysine residues (K) within a protein, resulting in an alteration of its activity. The opposite reaction is carried out by another group of enzymes historically called histone deacetylases (HDAC), which remove the acetyl moiety from target proteins. Sirtuins belong to class III HDAC, whose activity requires the coenzyme and sirtuin activator oxidized nicotinamide adenine dinucleotide (NAD^+^) for the deacetylation reaction. Hence, by deacetylating several important signaling proteins, they are supposed to play a key role in the regulation of glucose metabolism within cardiomyocytes [4,5,6]. NAD^+^ is an important metabolite involved in redox and energy homeostasis as well as in protein deacetylation reaction. Treatments with NAD^+^ precursors nicotinamide adenosine mononucleotide (NMN) and nicotinamide riboside (NR) reduce infarct size and induce cardioprotection. These effects are associated with reduced mitochondrial protein acetylation [7,8]. Therefore, balanced NAD^+^ recycling in cardiomyocytes is a critical determinant of sirtuins activity and therefore of their cardioprotective effects [9]. Understanding how NAD^+^ availability and protein acetylation regulate cardiac glucose and FA metabolism may have clinical relevance, as NAD^+^ precursors are already under investigation for cardiometabolic disorders and could represent potential therapeutic strategies.

**Figure 1:**
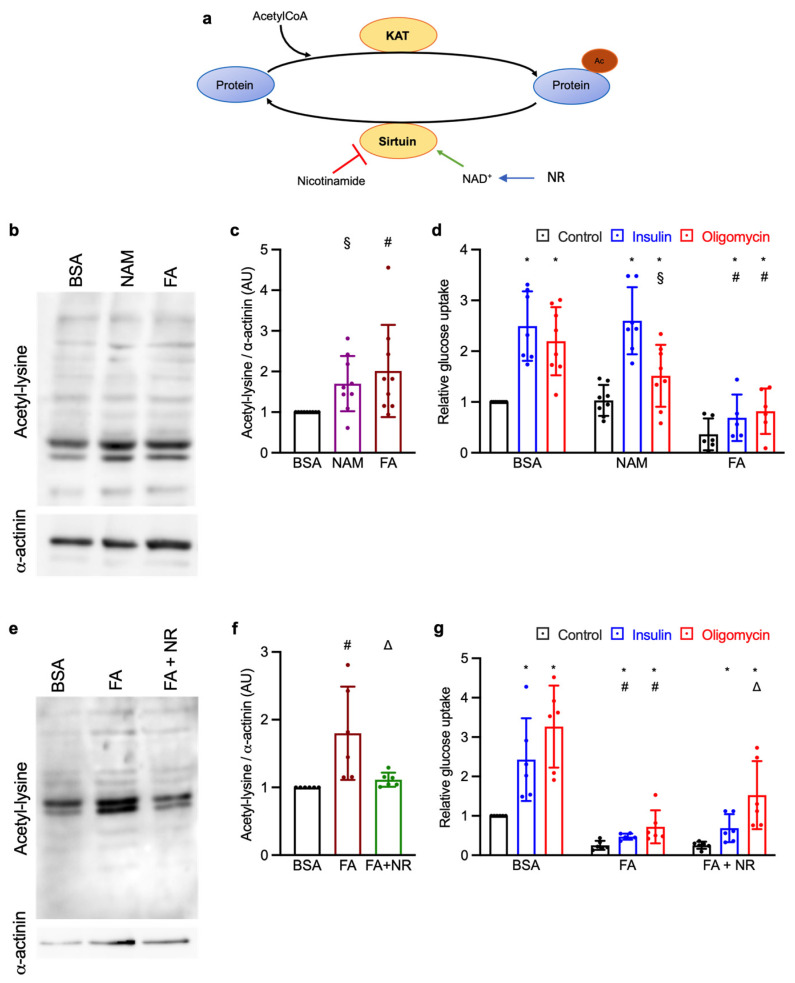
Effect of chronic NAM, FA, and NR treatment on lysine acetylation and glucose uptake in cardiomyocytes. (**a**) Diagram explaining why NAM or NR treatment should, respectively, increase or decrease protein lysine acetylation. (**b**–**g**): Primary rat cardiac myocytes were exposed for 7 days to control condition (BSA), NAM, FA, or FA + NR. (**b**–**d**) Effect of chronic NAM or FA exposure. (**b**) Representative Western blot of acetyl-lysine-containing proteins; α-actinin was used as a loading control. (**c**) Relative quantification of acetyl-lysine-containing proteins. (**d**) Basal and stimulated glucose uptake. (**e**–**g**) Effect of chronic NR treatment on glucose uptake in cardiomyocytes exposed to FAs. (**e**) Representative Western blot of acetyl-lysine-containing proteins; α-actinin was used as a loading control. (**f**) Relative quantification of acetyl-lysine-containing proteins. (**g**) Basal and stimulated glucose uptake. Relative glucose uptake was measured during 1 h exposure to either 1 μM insulin (blue bars), 1 μM oligomycin (red bars), or no stimulus (black bars). All values were normalized to the mean of the BSA control group, which was set to 1. Results are mean ± SD. *: significant (q < 0.05) effect of insulin or oligomycin; #: significant effect of FAs; §: significant effect of NAM; △: significant effect of NR.

We previously showed that chronic treatment with the phorbol ester TPA restores glucose uptake in cardiomyocytes exposed to FAs [10]. A preliminary metabolomic analysis showed that NAD^+^ was reduced in FA-treated cardiomyocytes, while chronic phorbol acetate treatment restored NAD^+^ levels to control condition. In this study, we investigate whether intracellular NAD^+^ reduction is responsible for increased protein acetylation and impaired glucose uptake induced by chronic FA exposure in cardiomyocytes. Therefore, we treated control cardiomyocytes with the sirtuin inhibitor nicotinamide (NAM) to replicate the effects of FA-induced lysine acetylation on glucose transport. Conversely, we replenished intracellular NAD^+^ concentration via chronic NR treatment in primary rat cardiomyocytes exposed to FAs (Figure 1a).

## 2. Materials and Methods

### 2.1. Rat Cardiomyocytes Culture

Male Sprague Dawley rats (100–200 g, Janvier Labs, Saint-Berthevin, France) were anesthetized by intraperitoneal administration of ketamine (100 mg/kg) combined with xylazine (10 mg/kg), and hearts were collected once deep anesthesia was achieved. All procedures complied with institutional and national ethical regulations and were approved by the Geneva Cantonal Committee for Animal Experimentation (permits #GE153). Experiments were performed following the ARRIVE guidelines.

Cardiomyocytes were isolated as previously described [11] by retrograde perfusion of the hearts with collagenase (type II; Worthington Biochemical, Lakewood, NJ, USA; 120 IU/mL) and hyaluronidase (1% *w*/*v*) [12,13]. Cardiomyocytes were separated from non-myocyte cardiac cells by pre-plating the whole cell suspension for 90 min on untreated plastic, to which non-myocyte cells, but not cardiomyocytes, readily adhere. Isolated cardiomyocytes were cultured in M199 medium (5.5 mM glucose) supplemented with creatine (5 mM), L-carnitine (2 mM), taurine (5 mM), cytosine-β-D-arabino-furanoside (100 μM), 9-cis retinoic acid (100 nM), triiodothyronine (10 nM), and 20% fetal bovine serum. Culture dishes were precoated with 0.1% gelatin for 4 h and incubated overnight with complete medium prior to cell plating. For imaging studies, cells were plated on laminin-coated coverslips. Fatty acids consisted of a 1:1 mixture of palmitate (C16:0) and oleate (C18:1 n-9) conjugated to bovine serum albumin, reaching a final concentration of 0.4 mM. NAM or NR was added at the time of cell plating. NAM was used at a final concentration of 10 mM as a sirtuin inhibitor; NR was used at a final concentration of 1 mM as a NAD^+^ precursor. The culture medium was renewed every 2–3 days, and subsequent analyses were performed on day 7. At this time point, control cardiomyocytes display a well-differentiated phenotype with stable insulin responsiveness [14].

### 2.2. Confocal Microscopy

Cardiomyocytes grown on laminin-coated coverslips were rinsed with ice-cold PBS, fixed in 4 mM paraformaldehyde for 20 min at room temperature, and fixation was quenched using 200 mM glycine. Cells were permeabilized with 0.3% Triton X-100 in PBS for 3 min, followed by blocking with 3% BSA and 0.1% Tween-20 to minimize non-specific antibody binding. For microtubules imaging, fixed and permeabilized cardiomyocytes were incubated overnight at 4 °C with a rabbit α-tubulin antibody (abcam #ab15246, Abcam Ltd., Cambride, UK), followed by secondary antibodies AlexaFluor 488-labeled F(ab′)2 goat anti-rabbit IgG (Invitrogen #A11070; Thermo Fisher Scientific, Waltham, MA USA). Following washes with PBS and H_2_O, coverslips were mounted on glass slides with ProLong Diamond antifade containing DAPI for DNA staining. Cardiomyocytes were examined with a Zeiss LSM800 confocal microscope (Carl Zeiss AG, Oberkofen, Germany), using a ×63 oil immersion objective. Confocal images were acquired as 0.32 µm optical sections across the entire cell thickness. Z-stack projections were reconstructed in ImageJ 1.8 using the maximum intensity projection setting. Brightness and contrast were uniformly adjusted with linear parameters across all experimental groups.

In cardiomyocytes, the nuclear envelope adopts the function of a cellular microtubule organizing center (MTOC) [15], which can be identified in microtubule imaging pictures as a perinuclear ring. MTOC organization was assessed based on the presence of a complete, partial or disrupted, or a not discernable perinuclear α-tubulin-labeled microtubule ring by two independent investigators in a blinded manner.

### 2.3. Glucose Uptake Measurement

Glucose uptake was assessed by uptake of [2,6-^3^H]-2-deoxyglucose (2-DG) as previously described [16]. Cardiomyocytes were incubated for 1 h at 37 °C in M199 medium containing 5.5 mM glucose and 10 nM [2,6-^3^H]-2-DG (1–2 μCi/mL, ANAWA Clinisciences Group, Zürich, Switzerland), with or without metabolic agonists. Insulin (10^−6^ M) and oligomycin (10^−6^ M, an F_O_-ATPase inhibitor inducing metabolic stress) were used as stimuli. Uptake was terminated by three PBS washes, followed by lysis in 1 mL of 0.1 M NaOH. Protein content was measured from two aliquots (20 μL each), while the remainder of the lysate was analyzed for radioactivity with a TriCARB 1900 TR scintillation counter (Packard Perkin-Elmer, Bridgeport, CT, USA). To allow statistical analysis over experiments obtained from separate cardiomyocytes isolations, in each experiment values were normalized to the mean of the BSA control group, which was set to 1.

### 2.4. Immunoblot Analysis

After insulin or oligomycin stimulation, the cells were washed three times with ice-cold PBS and lysed in 200 μL of buffer containing 150 mM NaCl, 50 mM Tris-HCl (pH 7.5), 1 mM EDTA, 0.5% sodium deoxycholate, 1% Igepal CA-630, and Halt protease/phosphatase inhibitors (Thermo Fisher Scientific, Waltham, MA, USA). Proteins (30 μg) from each sample were separated on SDS-PAGE gels and transferred onto polyvinylidene difluoride membranes. Primary and secondary antibodies used for Western blot analysis are listed in Appendix A. For all blots, incubation with the primary antibody was carried out overnight at 4 °C and incubation with the secondary antibody for 1 h at room temperature. Chemiluminescent signals were captured with an LAS-4000 imaging system (Fujifilm, Tokyo, Japan), and densitometric quantification was carried out using ImageJ. For each blot, intensity values were normalized so that the highest signal across treatments was set to 1.

### 2.5. Proteomic Analysis

Quantitative analysis of the lysine acetylome was performed using label-free quantitative (LFQ) approaches. Briefly, cultured cardiomyocytes were lysed, digested with trypsin, and the peptides analyzed by liquid chromatography–tandem mass spectrometry (LC-MS/MS) on an Orbitrap Fusion Lumos (Thermo Fisher Scientific, Waltham, MA, USA) mass spectrometer in line with an easy nLC1200 UHPLC (Thermo Fisher Scientific, Waltham, MA, USA). Data were generated by either data-independent acquisition (DIA) or data-dependent acquisition (DDA) mass spectrometry methods and using a chromatographic gradient of 120 min. Data analysis was performed using Spectronaut v.17 software (Biognosys, Schlieren, Switzerland), with the integrated PTM analysis workflow. Mass spectrometry data were generated by the Proteomics Core Facility of the Faculty of Medicine at the University of Geneva. Proteomic data included mass spectrometry data in .raw format, peak list files in .mgf format, search results files in .sne or .msf, as well as result export (.xlsx) and results report (.pdf).

No affinity enrichment for acetyl-lysine peptides was performed; thus, the acetyl-proteomics was used as a discovery-level screen rather than an exhaustive global survey. 

### 2.6. Pathway Analysis and Bioinformatics

Proteins showing significantly increased acetylation were ranked according to q-values and submitted to the g:Profiler tool (biit.cs.ut.ee/gprofiler/gost; accessed on 5 September 2024; Rattus norvegicus database). Ordered queries were run against KEGG and WikiPathways annotations restricted to ≤350 proteins to avoid overly broad categories. Multiple testing correction was performed using the g:SCS algorithm with a default α = 0.05.

### 2.7. Fatty Acid Uptake and Oxidation Measurements

Palmitate oxidation was determined from the rate of transfer of ^3^H from [9,10-^3^H]palmitate to ^3^H_2_O [17]. Cardiomyocytes were incubated for 60 min in a medium containing palmitate (0.05 mM), oleate (0.05 mM), and 1 μCi/mL [9,10-^3^H]palmitate (ANAWA Clinisciences Group, Zürich, Switzerland) complexed to bovine serum albumin (0.2 mM). After incubation, the medium (1 mL) was retrieved, immediately mixed with 1 mL of ice-cold 10% trichloracetic acid, and centrifuged at 2200× *g* for 10 min at 4 °C. The supernatant was neutralized with 250 µL of NaOH 6 M. ^3^H_2_O in the supernatant was separated from [9,10-^3^H]palmitate by anion exchange chromatography on Dowex 1 × 4. ^3^H_2_O eluted from the Dowex column was counted by liquid scintillation. 

Cardiomyocytes were washed twice with ice-cold PBS, dissolved in 0.1 M NaOH. Then, 20 µL aliquots were taken for protein content determination and the remaining NaOH lysate assayed for radioactivity in a TriCARB 1900 TR liquid scintillation analyzer (Packard). Palmitate uptake was calculated as the sum of intracellular ^3^H remaining in cells plus ^3^H_2_O released.

To allow statistical analysis over experiments obtained from separate cardiomyocytes isolations, in each experiment values were normalized to the mean of the BSA control group, which was set to 1.

### 2.8. NAD^+^ and NADH Determination

The cellular concentrations of NAD^+^ and NADH were measured in cultured cardiomyocytes with a colorimetric assay kit (Abcam #ab65348, Abcam Ltd., Cambridge, UK) according to the manufacturer’s instruction.

### 2.9. Statistics

Data are presented as mean ± SEM obtained from replicated experiments. Data were compared by one-way or two-way ANOVA (Prism 10, GraphPad Software) followed by post hoc testing for false discovery rates by the method of Benjamini and Yekutieli [18]. Post hoc testing indicated a positive discovery when the false discovery rate q was <0.05. MTOC scorings (Figure 3) were compared by a Chi-square test. Throughout the article, the following symbols are used for positive discoveries (q values): * indicates a significant effect of insulin or oligomycin stimulation as compared with unstimulated cardiomyocytes having received the same chronic treatment; # indicates a significant effect of chronic FA exposure, as compared with cardiomyocytes not exposed to FAs undergoing the same acute stimulation; § indicates a significant effect of chronic NAM exposure as compared with cardiomyocytes not exposed to NAM undergoing the same acute stimulation; △ indicates a significant effect of chronic NR exposure, as compared with cardiomyocytes not exposed to NR undergoing the same acute stimulation. 

## 3. Results

### 3.1. Effect of Chronic NAM and NR Treatments on Lysine Acetylation and Glucose Uptake

We started by testing the effect of different NAM concentration on lysine acetylation in cardiomyocytes not exposed to FAs. At high concentration (10 mM), NAM significantly increased lysine acetylation in the absence of FAs. In the same way, chronic FA exposure increased lysine acetylation in cardiomyocytes, as compared to the control group (Figure 1b,c). We next sought to evaluate whether chronic NAM treatment could influence the glucose uptake in the absence of FAs. NAM impaired metabolic-stress-stimulated glucose uptake in control cardiomyocytes, while no significant effect was observed on basal glucose uptake and upon insulin stimulation (Figure 1d). Chronic exposure to FAs markedly impaired insulin- and metabolic-stress-stimulated glucose uptake, which is consistent with our previous studies [10,19,20,21]. On the other hand, we sought to evaluate the effect of chronic NR treatment in FA-exposed cardiomyocytes. We assessed that NR (1 mM) restored NAD^+^ concentration in cardiomyocytes exposed to FAs (Figure S1). Indeed, we observed an increase in NAD^+^ and total NAD (NAD^+^ + NADH) concentrations as compared to the FA group, whereas the total NADH levels remained unaltered. We next evaluated whether this NAD^+^ replenishment by chronic NR supplementation could influence lysine acetylation and glucose uptake. Chronic NR treatment reduced lysine acetylation as compared to the FA-exposed cardiomyocytes (Figure 1e,f) and increased metabolic-stress-stimulated glucose uptake in FA-exposed cardiomyocytes (Figure 1g). Again, no effect was observed in the basal and insulin-stimulated conditions. Overall, these results demonstrate that FA-induced NAD^+^ reduction might be responsible for the increased lysine acetylation impaired glucose uptake in cardiomyocytes during metabolic stress.

### 3.2. NAM and NR Treatments Increase the AMPK Signaling Pathway

**Figure 2:**
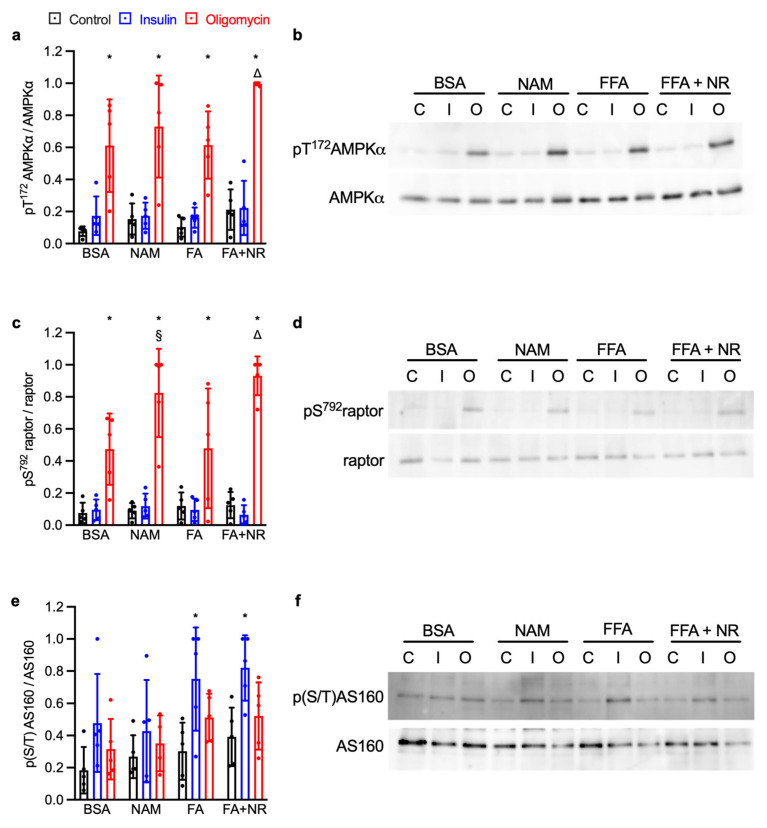
NAM and NR treatments increase the AMPK signaling pathway. Primary rat cardiac myocytes were exposed for 7 days to control condition (BSA), NAM, FA, or FA + NR. Cardiomyocytes were then stimulated for 10 min with 1 μM insulin (blue bars), for 20 min with 1 μM oligomycin (red bars), or left unstimulated (black bars). Relative phosphorylation of (**a**) T^172^ AMPK, (**c**) S^792^ raptor, and (**e**) AS160 on S/T residues. Results are mean ± SD. *: significant (q < 0.05) effect of insulin/metabolic stress; △: significant effect of NR; §: effect of NAM. (**b**,**d**,**f**) representative Western blots.

Since NAM and NR mainly affected glucose uptake under metabolic stress, we focused on AMP-activated protein kinase (AMPK), a key energy sensor regulating GLUT4 trafficking during ischemia-induced ATP depletion. AMPK activation was evaluated by phosphorylation of its α subunit at T^172^ and of its substrate raptor at S^792^. Oligomycin treatment significantly enhanced phosphorylation of both T^172^AMPKα (Figure 2a,b) and S^792^raptor (Figure 2c,d) across all experimental conditions. Both NAM and NR significantly increased T^172^AMPKα phosphorylation (Figure 2a,b) and S^792^raptor phosphorylation in cardiomyocytes (Figure 2c,d), as compared to the control and the FA group, respectively. The Rab GTPase-activating protein AS160, another substrate of AMPK, is known to regulate GLUT4 translocation. Its phosphorylation on multiple residues was detected using the S/T-phosphorylated Akt substrate antibody [22,23]. Neither NAM nor NR showed any effect on AS160 phosphorylation as compared to the control and FA group, respectively (Figure 2e,f). Thus, these results showed that the variations in glucose uptake induced by the various treatments impacting lysine acetylation are not explained by variations in AMPK activation.

### 3.3. Chronic NAM or FA Exposure Disrupts Microtubule-Organizing Centers (MTOCs)

**Figure 3:**
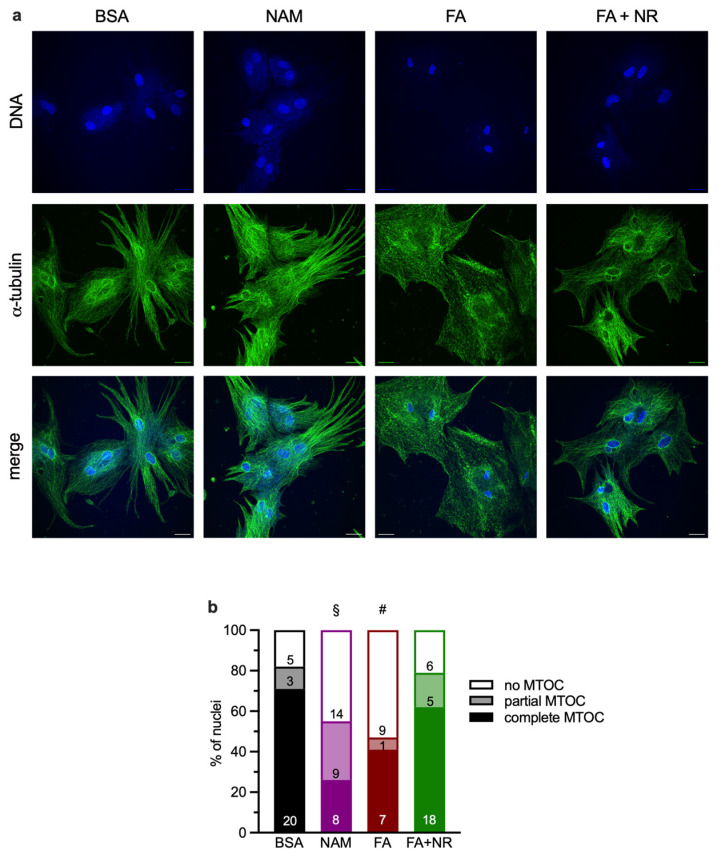
Effect of chronic NAM, FA and FA + NR treatments on microtubules and MTOC organization. Primary rat cardiac myocytes were exposed for 7 days to control condition (BSA), NAM, FA, or FA + NR. (**a**) Immunofluorescent images of tubulin (green) and nuclei (blue). Scale bar: 20 µm. (**b**) Scoring of MTOCs around nuclei as complete, partial, or absent. §: significant (*p* < 0.05) effect of NAM; #: significant effect of FAs. Number within columns represent total number or nuclei counted in three separate experiments.

Cytoskeletal organization, in particular microtubules and microtubule-organizing centers (MTOCs), has been shown to be important for glucose uptake [13,16]. In previous studies, we observed that microtubules organization was disrupted by chronic FA exposure in cardiomyocytes [10,19]. Cardiomyocytes in control condition displayed a well-organized microtubule network with prominent MTOCs; 71% of the nuclei examined exhibited complete MTOCs around them (Figure 3). NAM or FA exposure for 7 days resulted in a disrupted MTOC organization with 45% and 53%, respectively, of the nuclei examined totally lacking MTOCs. NR treatment of FA-exposed cardiomyocytes partially restored microtubules and MTOC organization, with 62% of the nuclei examined exhibiting a complete MTOC.

### 3.4. Proteomics Analysis Indicates HADHA Acetylation Is Influenced by Chronic NAM and NR Treatments

**Figure 4:**
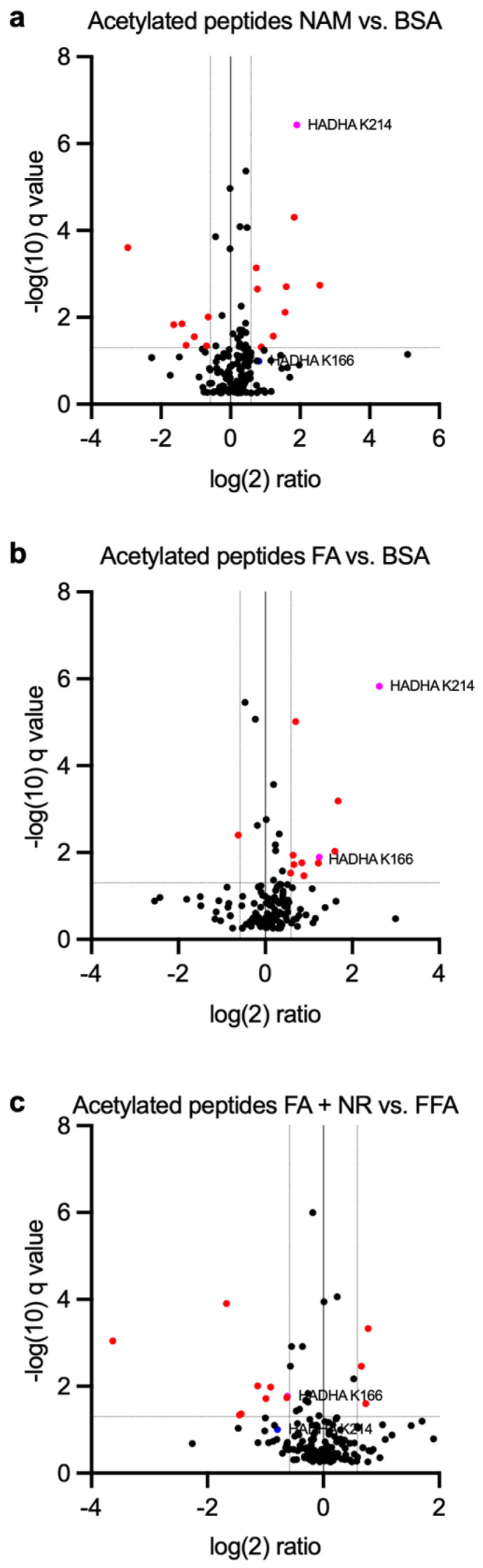
Proteomics analysis of the different lysine acetylation states. Primary rat cardiac myocytes were exposed for 7 days to control condition, NAM, FA, or FA + NR. (**a**–**c**) Volcano plots show significantly differentially acetylated proteins (red dots) in comparisons; (**a**) NAM vs. BSA; (**b**) FA vs. BSA; (**c**) FA vs. FA + NR. Peptides containing lysine residues K166 and K214 of HDAH are highlighted in magenta if significantly altered, and in blue if not.

Considering the discrepancies between NAM and NR treatments on AMPK signaling and cytoskeletal organization, we performed acetylomic analysis to identify possible target proteins that could be at the crossroad among the different conditions (Figure 4). LC-MS was performed on rat cardiomyocytes exposed to BSA, NAM, FA, or FA + NR treatments to assess the lysine acetylation state of the proteins. In this quantitative proteomic study, close to 1700 proteins were identified and quantified in these samples, and 166 acetylated sites (>75% of probability) were detected and quantified. Pathway enrichment analysis (g:Profiler) revealed that significantly acetylated proteins were mainly involved in the FA mitochondrial oxidation process (Figure S2). Among them, we detected that the hydroxyacyl-CoA dehydrogenase trifunctional enzyme subunit α (HADHA), a mitochondrial enzyme responsible for catalyzing the last three steps of fatty acid β-oxidation, had a variable lysine acetylation state among the conditions. Both chronic NAM and FAs induced HADHA hyperacetylation on residues K^166^ and K^214^ as compared to the control group. Conversely, NR treatment of cardiomyocytes exposed to FAs reduced HADHA K^166^ and K^214^ acetylation (Figure 4a–c and Table 1).

### 3.5. HADHA Acetylation Is Associated with Increased Fatty Acid Oxidation

**Figure 5:**
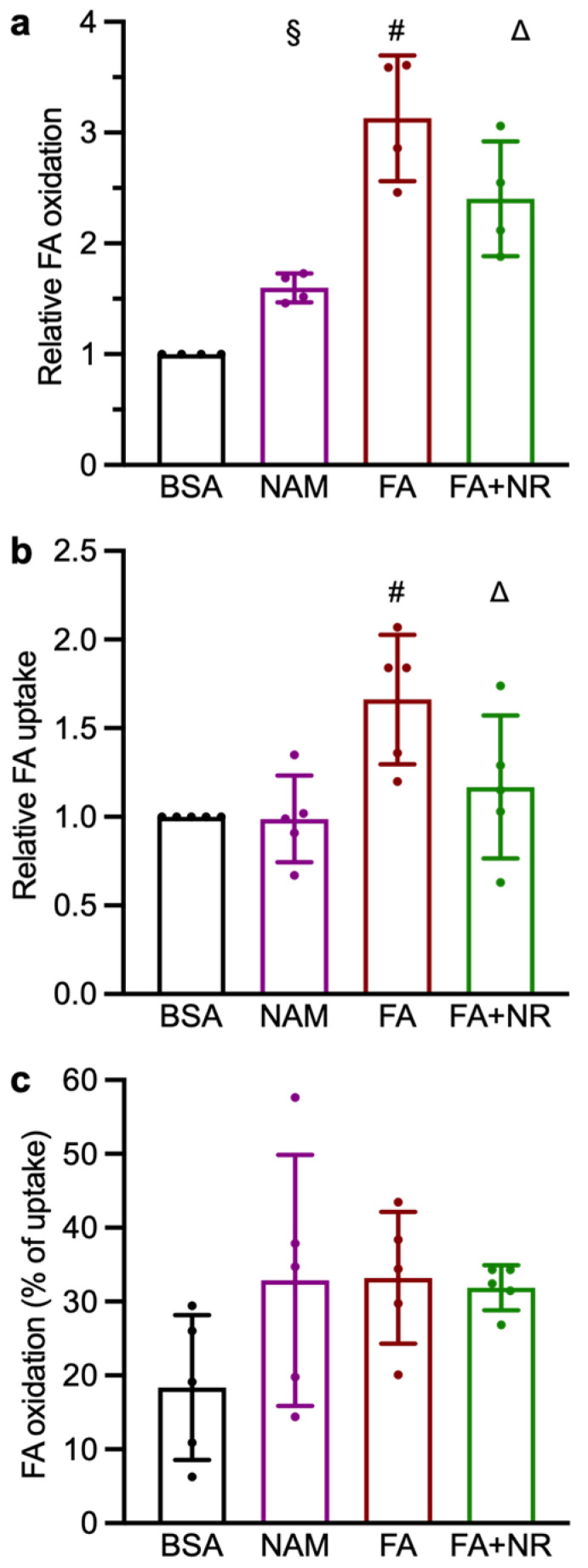
Effect of chronic NAM and NR treatments on palmitate uptake and oxidation in cardiomyocytes. Primary rat cardiac myocytes are exposed for 7 days to control condition (BSA), NAM, FA, or FA + NR. (**a**) Effect of chronic NAM and NR treatments on palmitate uptake. (**b**) Effect of chronic NAM, FA, and FA + NR treatments on palmitate oxidation. (**c**) Effect of chronic NAM, FA, and FA + NR treatments on palmitate oxidation expressed as a percentage of the uptake. All values were normalized to the mean of the BSA control group, which was set to 1. Results are mean ± SD; §: significant effect of NAM (q < 0.05); #: significant effect of FAs; △: significant effect of NR.

As mentioned earlier, the trifunctional enzyme subunit α (HADHA) plays a crucial role in fatty acid oxidation. Therefore, we investigated whether lysine acetylation could impact fatty acid metabolism by measuring palmitate uptake and oxidation (Figure 5). We observed that both NAM and FAs, which represent conditions with increased HADHA K^166^ and K^214^ acetylation, increased palmitate oxidation as compared to the control (Figure 5a). In contrast, only the FA treatment increased palmitate uptake (Figure 5b). On the other hand, chronic NR treatment reduced both palmitate uptake and oxidation in FA-exposed cardiomyocytes (Figure 5a,b). The oxidation of palmitate expressed as a percentage of the palmitate taken up showed a tendency to be increased in all three treatment groups as compared to the control (Figure 5c).

## 4. Discussion

During the 1960s, Randle and colleagues demonstrated that FAs have the ability to inhibit glucose metabolism, in a regulatory mechanism commonly referred to as the “Randle cycle” or the “glucose–FA cycle” [24]. Over the course of several decades, studies have revealed that FAs inhibit glucose utilization at various stages, including glucose uptake, glycolysis, and mitochondrial oxidation. While the molecular mechanism governing glycolysis and glucose oxidation are well defined, the impact of FAs on glucose transport remains poorly understood [25]. Recent evidence suggests that protein acetylation could play a crucial role in cardiac metabolic inflexibility [26,27,28]. Hence, investigating the role of protein acetylation in the context of diabetic cardiomyopathy could provide new therapeutic opportunities for restoring cardiac metabolic flexibility. One approach could involve countering the increase in protein acetylation by inhibiting KATs or implementing strategies to replenish NAD^+^ levels and enhance the activity of sirtuin deacetylases.

In the present study, we investigated whether intracellular NAD^+^ reduction is responsible for increased protein acetylation and impaired glucose uptake induced by chronic FA exposure in cardiomyocytes. To address this, we aimed to increase the NAD^+^ concentration in cardiomyocytes chronically exposed to FAs using the NAD^+^ precursor NR. Conversely, we inhibited sirtuin activity (NAD^+^-dependent deacetylases) in cardiomyocytes in the absence of FAs to understand if we could replicate the detrimental effect of FAs on cardiac glucose transport induced by NAD^+^ reduction and increased lysine acetylation. The first point to be discussed is the pharmacokinetics of NAM as a sirtuin inhibitor. Both NAM and NR are NAD^+^ precursors, which follow different metabolisms to be converted into NAD^+^. NR is converted to nicotinamide mononucleotide (NMN) by the NR kinase (NRK), while NAM is converted into NMN by the NAM phosphotransferase (NAMPT) in the salvage pathway [29]. For this reason, both of them have been used to increase NAD^+^ biosynthesis in preclinical studies on cardiovascular disorders [30,31,32,33,34,35]. On the other hand, during sirtuin deacetylation reaction, NAM derived from the cleavage of one molecule of NAD^+^ will act as an allosteric sirtuin inhibitor [36]. Therefore, whether NAM acts as sirtuin inhibitor or activator as a NAD^+^ precursor seems to depend on the different type of cells, disorders, and duration of the treatment. To our knowledge, no other study has used NAM chronically as a sirtuin inhibitor in primary rat cardiomyocytes. In our study, we tested different NAM concentrations to assess its effect on lysine acetylation, and we observed that NAM increased lysine acetylation only at a concentration higher than 5 mM. 

One of the main findings of this study regards the effect of NAM and NR on lysine acetylation and glucose uptake. We observed that chronic NAM treatment increased lysine acetylation and impaired metabolic-stress-stimulated glucose uptake in the absence of FAs. On the other hand, chronic NR supplementation reduced lysine acetylation and improved metabolic-stress-stimulated glucose uptake in FA-exposed cardiomyocytes. These results are consistent with other studies indicating the importance of sirtuin activity and NAD^+^ availability during I/R. Increasing the NAD^+^ concentration has been shown to increase SIRT1 and SIRT3 activity, protecting the heart during I/R. In contrast, NAD^+^ precursors failed to reduce infarct size upon sirtuin downregulation, showing that the positive effect of NAD^+^ was mediate via sirtuin activation [4,5,6,8].

We also observed that our treatments influenced only metabolic-stress-stimulated glucose uptake, whereas no effect was observed on the response to insulin. This leads to further considerations: (1) Acetylation is a post-translational modification resulting from the excessive FA oxidation, which increases acetyl-CoA production and reduces NAD^+^ levels. Therefore, reduced NAD^+^ levels are not the only determinant regulating the acetylation process. Inhibition of lysine acetyltransferase (KAT) activity prevents FA-induced lysine acetylation and preserves insulin-stimulated glucose transport [37]. (2) The deacetylation reaction is not only mediated by sirtuin activity, since other NAD^+^-independent deacetylases (HDAC) are present in the heart. Inhibition of HDAC6 has been shown to impair insulin-stimulated glucose uptake in cardiomyocytes [38]. Therefore, one might speculate that insulin-stimulated glucose uptake might not be affected by sirtuin-mediated deacetylation.

Activation of the AMPK complex mediates the stimulation of myocardial glucose uptake by metabolic stress [20,21,39,40]. Thus, we investigated AMPK signaling, assessed at three levels: phosphorylation of AMPKα on T^172^, phosphorylation of raptor (an AMPK substrate not involved in the regulation of glucose uptake) on S^792^, and phosphorylation of AS160 on T and S residues. We have found that regardless of the effect observed on glucose uptake, NAM and NR increased both T^172^ AMPKα and S^792^ raptor phosphorylation, the latter used as a readout for AMPK activity. Raptor phosphorylation was previously shown to better correlate with metabolic-stress-stimulated glucose uptake than AMPKα phosphorylation [20]. However, as far as we know, raptor is not directly involved in the regulation of glucose uptake in cardiomyocytes [41]. In contrast to raptor, the phosphorylation of AS160 is known to be required for translocation of GLUT4 to occur in response to metabolic stress, and AS160 is a direct target of AMPK [23,42]. Indeed, the variations in AS160 phosphorylation generally matched the changes in AMPKα phosphorylation upon oligomycin stimulation. However, NAM- and FA-related variations go in different directions as compared to glucose uptake, showing that NAM- and FA-induced protein acetylation might affect mechanisms of GLUT4 translocation downstream of AS160 or separate from the AMPK signaling pathway.

Cytoskeletal integrity and plasticity also play an important role in the translocation of the GLUT4-containing vesicle to sarcolemma in response to stimuli in cardiomyocytes [13,43]. Herein, we observed a good correlation between microtubules and MTOC organization and responsiveness of glucose uptake to metabolic stress. In a previous study, we observed a link between MTOC preservation and efficient glucose transport in cardiomyocytes; inhibition of the ERK1/2 MAP kinases was shown to improve glucose transport and to preserve MTOCs [16]. However, in this study, intact microtubular structure also preserved the glucose uptake in response to insulin stimulation [16], whereas in our study, the insulin stimulation was not restored when MTOCs were restored by NR treatment of FA-exposed cardiomyocytes. This suggests that well-organized MTOCs are necessary for proper stimulation of glucose uptake by either stimulus; however, in the case of insulin-stimulated glucose uptake impaired by FA exposure, MTOC restoration is not sufficient.

One key finding of this study involves the identification of a target protein that potentially plays a crucial role among the conditions, serving as a point of convergence. The trifunctional enzyme subunit α (HADHA) catalyzes the last three steps of FA β-oxidation. Increased HADHA acetylation has been associated with enhanced FA oxidation and increased acetylation of mitochondrial protein in the diabetic heart [44]. Whether NAD^+^ replenishment induces deacetylation of HADHA remains unknown. In an acetyl-proteomic screening, we discovered that two HADHA lysine residues (K^166^ and K^214^) are acetylated upon chronic FA and NAM treatment, and deacetylated in FA + NR-treated cardiomyocytes. Acetylation of HADHA K^166^ and K^214^ residues correlated with increased FA oxidation, while NR treatment of FA-exposed cardiomyocytes reduced both HADHA K^166^ and K^214^ acetylation and FA oxidation [45]. To the best of our knowledge, acetylation of K^166^ and K^214^ had not yet been associated with any regulation of FA oxidation. Overall, these findings suggest that NAD^+^-dependent deacetylation might play a role in the regulation of metabolic enzymes activity, thereby influencing FA and glucose oxidation in cardiomyocytes. In addition to regulating fatty acid oxidation, protein acetylation has also been implicated in controlling pyruvate import into mitochondria, thereby influencing TCA cycle fueling and oxidative phosphorylation [26,46]. As there exists indication of a positive feedback loop from pyruvate oxidation to glucose uptake in cardiomyocytes [47], we cannot exclude that a reduction in pyruvate oxidation as a result of impaired pyruvate import into mitochondria would result in reduced glucose uptake. However, one would expect such an effect to manifest also in the response to insulin, not only in that to oligomycin. 

Some limitations of this study deserve comments. Due to technical reasons, the primary limitation of this study was the low number of replicates used for mass-spectrometry-based analysis. Quantitative analysis highlighted few peptides which modified acetylation state upon treatment. Indeed, adding the acetylation modification to the search parameters allowed us to identify 166 acetylation sites with at least 75% probability on 97 distinct proteins in these samples. As expected, as no enrichment was performed beforehand, the number of identified acetylated peptides, mostly mitochondrial, was rather low. Nevertheless, a clear signal was obtained, and we were able to identify a target protein (HADHA) whose lysine acetylation state was variable among treatments.

The second limitation was the use of the ATPase inhibitor oligomycin as a surrogate for ischemia in vitro. Ischemia in vivo impacts the mitochondrial function in a much more complex manner than simply shutting off ATPase activity. However, simulated ischemia in vitro, achieved with glucose and oxygen deprivation, takes a much longer time to act than in vivo, long enough that changes in protein expression, such as overexpression of GLUT1, can occur and confound the interpretation of glucose uptake data [48]. Therefore, oligomycin remains a convenient way to achieve ischemia-like energy depletion and AMPK activation without changing protein expression.

## 5. Conclusions

**Figure 6:**
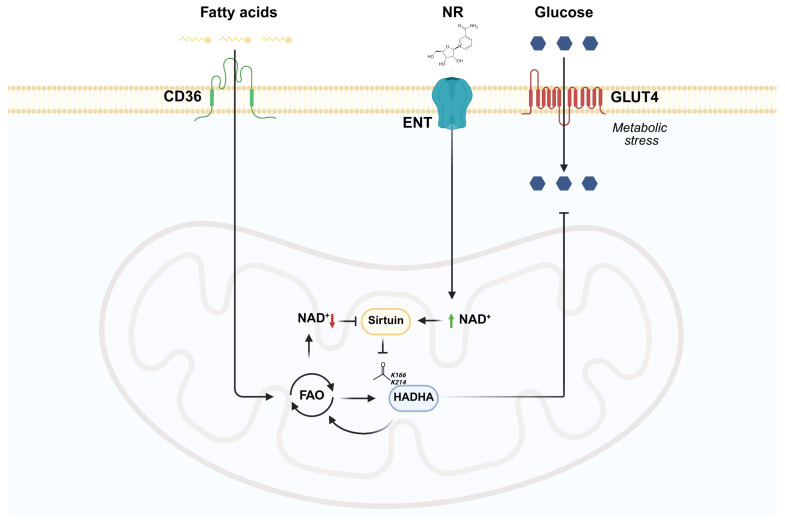
Conclusion. In cardiomyocytes chronically exposed to FAs, HADHA acetylation on K^166^ and K^214^ is associated with reduced NAD^+^ levels and increased FA oxidation (FAO). Conversely, replenishing the NAD^+^ concentration with NR reduces HADHA acetylation and is associated with reduced FAO and improved glucose uptake under metabolic stress. Created with BioRender.com/w66c655.

We showed that NAD^+^ replenishment reduced lysine acetylation and improved glucose uptake during metabolic stress in FA-exposed cardiomyocytes. However, it is worth noting that we did not observe any significant effects on insulin stimulation. We found that chronic FA exposure increased FA oxidation together with increased acetylation of HADHA on K^166^ and K^214^. On the other hand, NAD^+^-dependent HADHA deacetylation was associated with reduced FA utilization in FA-exposed cardiomyocytes (Figure 6). These findings suggest that modulation of NAD^+^ metabolism and lysine acetylation may provide a potential therapeutic avenue to restore metabolic flexibility and protect the diabetic heart during ischemia.

## Figures and Tables

**Table 1:** Differential lysine acetylation of HADHA.

	NAM vs. BSA	FA vs. BSA	FA + NR vs. BSA
HADHA K^166^	Fold change 2.35 q = 0.102	Fold change 2.36 q = 0.0129	Fold change 0.654 q = 0.0170
HADHA K^214^	Fold change 3.76 q = 3.67 × 10^−7^	Fold change 6.15 q = 1.49 × 10^−6^	Fold change 0.578 q = 0.099

## Data Availability

The complete acetyl-proteomics data obtained are available as Supplementary Data to this study. Other datasets generated during and/or analyzed during the current study are available from the corresponding author on reasonable request.

## Supplementary Materials

The following supporting information can be downloaded at: https://www.mdpi.com/article/10.3390/metabo15100636/s1. Figure S1: Effect of chronic FA and NR treatments on NAD+ and NADH content in cardiomyocytes. Figure S2: Pathway characterization of cardiac proteins displaying increased acetylation. Supplementary files: complete proteomics and acetylomics data files: pl23023_EVanni_allQuantified_AcPept.xlsx; pl23023_EVanni_allQuantified_Proteins.xlsx; original confocal immunofluorescence acquisition data: BSA.czi; NAM.czi; FA.czi; FA + NR.czi; Original images.pdf.

## Abbreviations

The following abbreviations are used in this manuscript:

AMPK	AMP-activated protein kinase
ANOVA	Analysis of variance
BSA	Bovine serum albumin
EDTA	Ethylenediaminetetraacetic acid
FA	Fatty acid
HADHA	Hydroxyacyl-CoA dehydrogenase trifunctional enzyme subunit α
HDAC	Histone deacetylase
KAT	Lysine acetyl transferase
MTOC	Microtubule-organizing center
NAD^+^/NADH	Nicotinamide adenine dinucleotide (oxidized/reduced)
NAM	Nicotinamide
NR	Nicotinamide riboside
PBS	Phosphate-buffered saline

## Appendix A

**Table A1:** Primary and secondary antibodies used in Western blots.

Antigen	Conjugation	Company	Catalog Number
*Primary antibodies*			
Acetylated-lysine		Cell Signaling Technology (Leiden, The Netherlands)	9441
AMPKα		Cell Signaling Technology	2532
AMPKα; phosphoT172		Cell Signaling Technology	2535
AS160		Cell Signaling Technology	2670
Phospho-(Ser/Thr) Akt substrate		Cell Signaling Technology	9611
raptor		Cell Signaling Technology	2280
raptor; phosphoS792		Cell Signaling Technology	2083
α-actinin		Sigma-Aldrich (Buchs, Switzerland)	A7811
*Secondary antibodies*			
Rabbit IgG	Horse radish peroxidase	Cell Signaling Technology	7074
Mouse IgG	Horse radish peroxidase	Cell Signaling Technology	7076
