# Supplementary material for: NAD+-Dependent Lysine Acetylation Regulates Glucose Uptake and Fatty Acid Oxidation in Cardiomyocytes
Source: Metabolites. 2025 Sep 23;15(10):636. doi: 10.3390/metabo15100636 (PMC12565917; doi:10.3390/metabo15100636)
Supplement: Supplementary file 1 [file metabolites-15-00636-s001.zip › Supplementary material R1.pdf]

Supplementary material

Supplementary Figure S1. Effect of chronic FA and NR treatments NAD^+ and NADH content in cardiomyocytes. Primary rat cardiac myocytes are exposed for 7 days to control condition (BSA), FA, or FA + NR. Relative concentrations of NAD^+ (a), NADH (b) and total NAD (NAD^+ + NADH; c). All values were normalized to the mean of the BSA control group, which was set to 1. Results are mean ± SD; #: significant ($q < 0.05$) effect of FA; △: significant effect of NR.

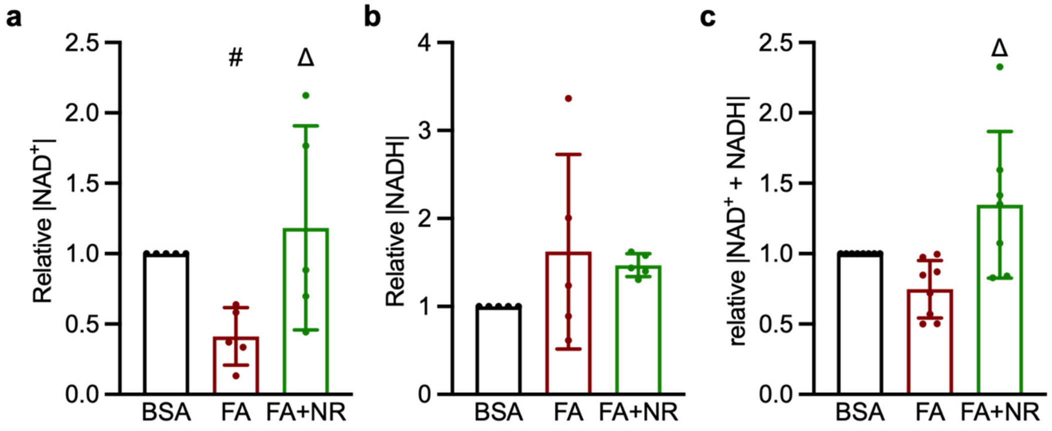

Supplementary Figure S2: Pathways characterization of cardiac proteins displaying increased acetylation. Functional enrichment analysis of over-represented KEGG pathways in FA vs. BSA, NAM vs. BSA, and FA + NR was performed using g:Profiler. Proteins were imputed in g:Profiler in ordered query, with decreasing q value and threshold set at q < 0.05. The maximum size of functional categories was set at 350 to filter out large annotations providing limited interpretative value. The g:SCS algorithm was used for multiple hypothesis testing corrections (q < 0.1). Enrichment is expressed as the number of proteins observed for a given pathway term.

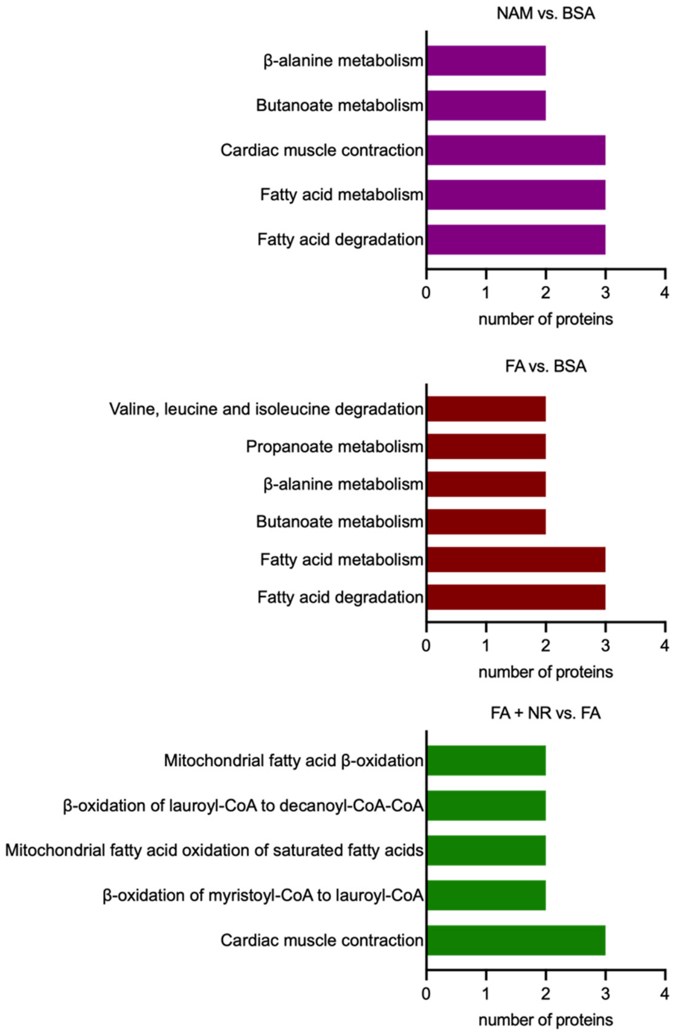

Supplementary files: complete proteomics and acetylomics data files:

pl23023_EVanni_allQuantified_AcPept.xlsx

pl23023_EVanni_allQuantified_Proteins.xlsx

Supplementary files: original confocal immunofluorescence acquisition data:

BSA.czi

NAM.czi

FA.czi

FA+NR.czi

Supplementary file: Original uncropped western blots and immunofluorescence images

Original images.pdf
