# Supplementary figures and images for: NAD+-Dependent Lysine Acetylation Regulates Glucose Uptake and Fatty Acid Oxidation in Cardiomyocytes
Source: Metabolites. 2025 Sep 23;15(10):636. doi: 10.3390/metabo15100636 (PMC12565917; doi:10.3390/metabo15100636)

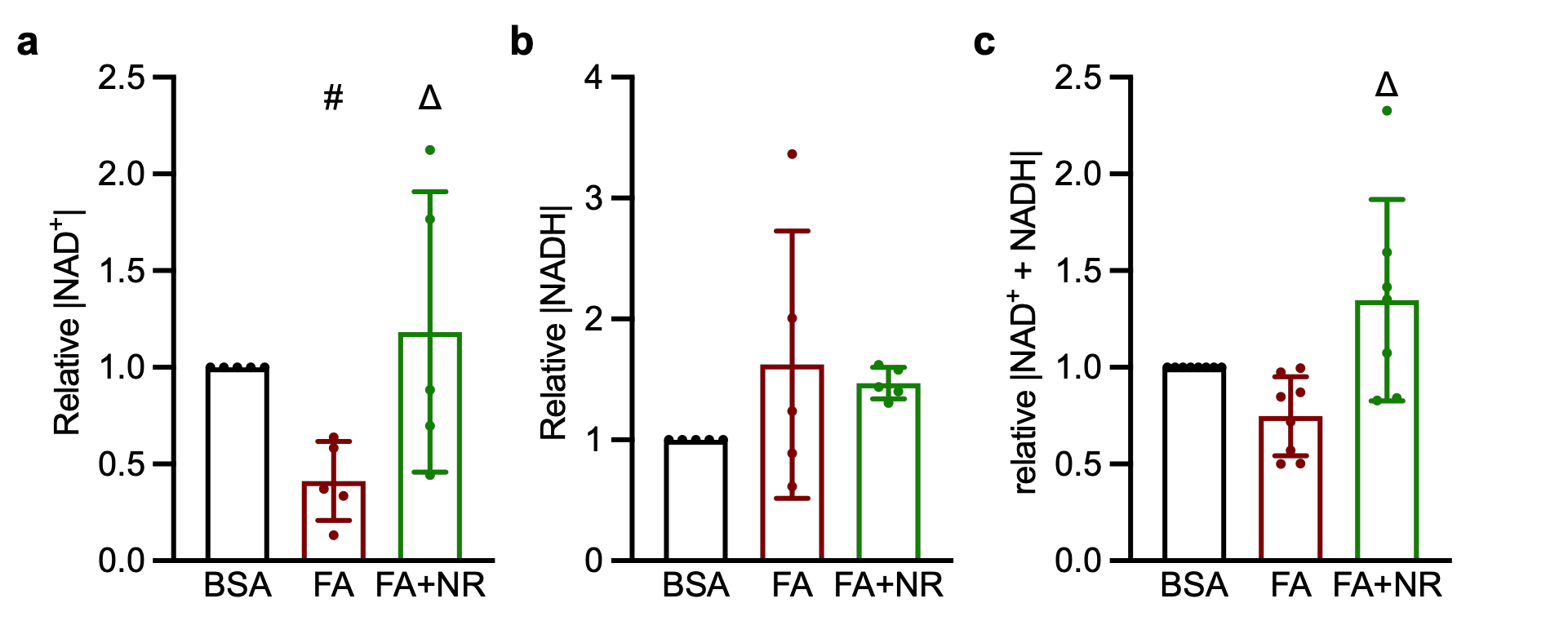

Supplement: Supplementary file 1 [file metabolites-15-00636-s001.zip › S1 Metabolites R1.tiff]

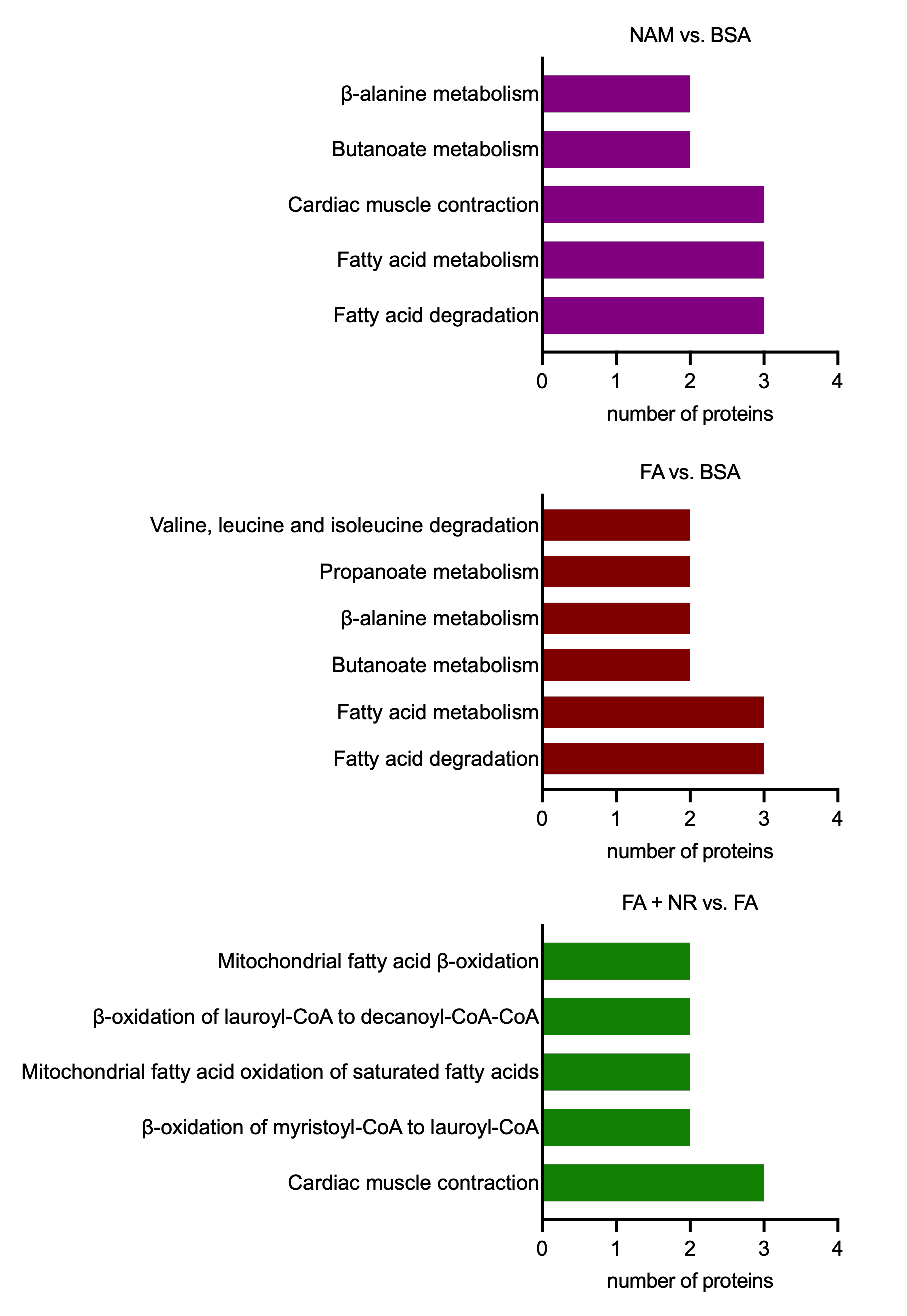

Supplement: Supplementary file 1 [file metabolites-15-00636-s001.zip › S2 Metabolites R1.tiff]
